# Hepatic Gene Expression Profiles Are Altered by Dietary Unsalted Korean Fermented Soybean (Chongkukjang) Consumption in Mice with Diet-Induced Obesity

**DOI:** 10.1155/2011/260214

**Published:** 2011-03-09

**Authors:** JuRyoun Soh, Dae Young Kwon, Youn-Soo Cha

**Affiliations:** ^1^Department of Food Science and Human Nutrition, and Research Institute of Human Ecology, Chonbuk National University, 664-14 Dukjin-Dong 1-Ga, Jeonju 561-756, Republic of Korea; ^2^Food Functional Research Division, Korean Food Research Institutes, San 46-1, Baekhyun-dong, Bundang-gu, Sungnam-si, Gyeonggi-do 463-746, Republic of Korea

## Abstract

We found that Chongkukjang, traditional unsalted fermented soybean, has an antiobesity effect in mice with diet-induced obesity and examined the changes in hepatic transcriptional profiles using cDNA microarray. High-fat diet-induced obese C57BL/6J mice were divided into three groups: normal-diet control group (NDcon, 10% of total energy from fat), high-fat diet control group (HDcon, 45% of total energy from fat), and HDcon plus 40% Chongkukjang (HDC) and were fed for 9 weeks. The HDC group mice were pair-fed (isocalorie) with mice in the HDcon group. Final body weight, epididymal fat accumulation, serum total cholesterol, and LDL-cholesterol were improved in HDC group. The cDNA microarray analyses revealed marked alterations in the expression of about 800 genes. Several genes involved in fatty acid catabolism (Acaa2, Mgll, Phyh, Slc27a2, and Slc27a5) were normalized by Chongkukjang consumption. This study showed beneficial effects of Chongkukjang consumption in preventing diet-induced obesity and related metabolic abnormalities.

## 1. Introduction

The incidences of various chronic diseases and diseases of aging in industrialized societies are increased by excess energy intake and lack of exercise. One example is metabolic syndrome, characterized by obesity, insulin resistance, glucose intolerance, dyslipidermia, and hypertension. Numerous studies suggest that excessive energy intake is the most fundamental cause of obesity, and high-energy intake is not only related to the absolute amount of lipid but also to the energy density of food [1–3]. Therefore, in planning meals to reduce obesity, it is ideal to reduce energy density by reducing fat and increasing fiber while maintaining suitable protein intakes.

It has been reported that beans modulate or prevent chronic and acute diseases of adults, including hardening of the arteries, heart disease, diabetes, senile dementia, cancer, and osteoporosis [4]. Soybean proteins also have stronger antioxidative properties than casein against lipid oxidation [5], and they have also been demonstrated to lower blood cholesterol and triacylglycerols [6].

Chonkukjang is prepared by cooking soybeans and inoculating with microorganism to initiate fermentation. Chonkukjang has distinct characteristics when compared to other fermented soybean products. It is fermented predominantly with *Bacillus subtilis* for short periods (2 days) without salts or other seasonings. During fermentation, isoflavonoids are converted from glycosides into the corresponding aglycones. The amount of daidzein in fermented soybean significantly increased by 44-fold, compared with those in unfermented soybean [7], and most proteins are degraded into small peptides and amino acids [8, 9]. Due to the fermentation process, Chonkukjang has additional physiological effects, in addition to those intrinsic to the soybeans themselves [10]. These reported functions include thrombus dissolution, improving vein inner-layer function, hypotensive effects, improvement of lipid profiles, antimutagenesis, anticancer, and antifungal effects [11]. However, there is a lack of research into the potential antiobesity effects of Chonkukjang intake.

Therefore, in this study, we investigated the effects of Chonkukjang consumption on weight gain, epididymal fat accumulation, lipid metabolism, and hepatic mRNA expression in mice with diet-induced obesity. To obtain a more comprehensive picture of the diet-induced hepatic transcriptional adaptation in the C57BL/6J mouse, we used cDNA microarray, containing ~10,000 mouse transcripts.

## 2. Materials and Methods

### 2.1. Experimental Animal and Diets

Male 4-week-old C57BL/6J mice were fed a chow diet for 1 week and then divided into 3 groups (*n* = 10) by randomized block design. During the experimental periods, a modified AIN-93 diet (Tables 1(a) and 1(b)) was supplied as pellet. NDcon (normal-diet control group, 10% of total energy from fat) and HDcon (high-fat diet control group, 45% of total energy from fat) were given free access to diet and water, and the HDC (60 g high-fat diet (60% of total energy from fat) plus 40 g Chongkukjang-supplemented group) mice were pair fed (isocalorie) with those in the HDcon group. After being maintained on their respective diets for 9 weeks, animals were sacrificed after fasting for 12 hours. Dietary intake and weight were recorded every other day and weekly, respectively. Room temperature and humidity were maintained at 23 ± 1°C and 53 ± 2%, respectively, with a 12 hours light/dark cycle. This study was conducted in conformity with the policies and procedures of the Institutional Animal Care and Use Committee of the Chonbuk National University. Based on the AIN-93 modified diet (Research Diet. Inc New Brunswick, NJ, USA), fat content of the high-fat diet was 24% (45% of total calories from fat), and fat content of the normal diet was 4% (10% of total calories from fat). Chongkukjang was prepared by a traditional processing method at the Department Food Science and Technology of Chonbuk National University (Jeonju, Korea) and mixed with the high-fat diet. Soybeans were sorted, washed, and soaked in water for 12 hours at +15°C and boiled for 4 hours at +100°C. The cooked soybeans were cooled to +40°C and fermented predominately with *Bacillus subtilis* in a fermentation chamber at +30°C for 43 hours.

### 2.2. Sample Collection and Analysis

To reduce the effects of differences in dietary intake, blood samples were collected after 12 hours overnight fasting by orbital venipuncture. Blood samples were left on ice for one hour and centrifuged at 1100 ×g for 15 minutes, and serum was separated and collected. Serum samples were stored at −80°C before analysis. After blood collection, liver and epididymal fat were surgically removed, washed in saline to remove foreign substances, dried on filter paper, and quick frozen in liquid nitrogen after weighing.

### 2.3. Analysis of Lipids

Serum triglyceride, total cholesterol, and HDL-cholesterol levels were estimated by an enzymatic colorimetric method using a commercial assay kit (Asan Pharm. Co, Seoul, Korea). LDL-cholesterols were analyzed by the Friedwald method [LDL-cholesterol = total cholesterol − HDL-cholesterol − (triglyceride)/5] [12]. 

### 2.4. Microarray Analysis

Mouse 10K cDNA microarray used in this study consisted of 10336 spots. It included 6531 transcripts from National Institute of Aging (NIA), 1243 transcripts from Brain Molecular Anatomy Project (BMAP), 2060 transcripts from InCyte Pharmaceuticals (Fremont, CA, USA), and yeast DNA and housekeeping genes as negative controls. Total RNA was prepared from livers using Trizol (Invitrogen, Carlsbad, CA, USA). Fluorescence-labeled cDNA probes were prepared from 20 *μ*g of total RNA by using amino-allyl cDNA labeling kit (Ambion, Austin, Texas, USA). Equal amounts of the RNA from 6 mice of each group were mixed, and each sample was equally divided; one half was used to generate Cy3-labeled cDNA, the other half was used to generate Cy5-labeled cDNA for dye swapping. The Cy5 and Cy3 probes were mixed, and hybridization was performed at +55°C for 16 hours, as previously described [13].

The two fluorescent images (Cy3 and Cy5) were scanned separately by a GMS 418 Array Scanner (Affymetrix, Santa Clara, CA, USA), and the image data were analyzed using ImaGene 4.2 (Biodiscovery, Santa Monica, CA, USA) and MAAS (Gaiagene, Seoul, Korea) softwares [14]. For each hybridization, emission signal data were normalized by multiplying the Cy3 signal values by the ratio of the means of the Cy3 and Cy5 signal intensities for all spots on the array. To eliminate the unreliable data, the following criteria were adopted. (1) PCR amplification of the sequence spotted on the array was deemed acceptable only if the amplification was confirmed and a single size product was obtained. (2) Accurate printing of each spot was required, as shown by emission signal from more than 40% of the spot area. (3) The signal from the fluorophore labels had to be higher than 256 (2^8^). 

### 2.5. Real-Time RT-PCR

4 *μ*g of total RNA were reverse transcribed in 25 *μ*L of reaction mixture, containing MuLV reverse transcriptase (2.5 unit), RNase inhibitor (1unit), 5 mmol/L MgCl_2_, 50 mmol/L KCl, 10 mmol/L Tris-HCl, (pH 8.3), 2.5 *μ*mol/L oligo (dT) primer, and 1 mmol/L dNTPs. The reaction mixture was heated to +42°C for 60 minutes and then denatured at +85°C for 5 minutes. cDNA was amplified with ICycler (BioRad, Hercules, CA, USA) in 50 *μ*L of reaction mixture containing AmpliTaq DNA polymerase (1 unit, Perkin Elmer, Shelton, CT, USA), 50 mmol/L Tris (pH 8.3), 0.25 g/L BSA, 3 mmol/L MgCl_2_, 0.25 mmol/L dNTPs, 1/50,000 dilution of SYBR green I (Molecular Probes, Eugene, OR, USA), and 0.25 *μ*mol/L appropriate forward and reverse PCR primers. PCR was performed with the primers indicated in Table 2. The following cycling conditions were used: one denaturing cycle at +95°C for 5 minutes, followed by 30 cycles of +95°C for 30 sec, +60°C for 45 sec, and +72°C for 1 minute. Relative RNA levels were determined by analyzing the changes in SYBR green I fluorescence during PCR according to the manufacturer's instructions. *β*-actin was amplified in parallel, and the results were used for normalization. The correct size of PCR products was confirmed by electrophoresis on a 2% agarose gel stained with ethidium bromide. Purity of the PCR products was determined by melting point analysis, using the ICycler software.

### 2.6. Statistical Analysis

Mice data from individual experiments were expressed as the mean ± standard deviation. SAS version 8 (SAS Institute, Cary, NC USA) was used for all statistical analyses. Significance of differences were analyzed by Duncan's multiple rage test, and the accepted level of significance was *P* < .05. In the microarray analysis, we calculated the median gene expression ratios from six independently repeated microarray experiments. We used the SAM method for multiclass response data to test whether differences in gene expression were significant [15]. We took the genome-wide significance level at the SAM(*δ*) = 0.66 and adopted a cutoff of 2.0-fold change based on our experiences. 

## 3. Results

### 3.1. Effects of Chongkukjang on Weight-Related and Biochemical Parameters

**Figure 1:**
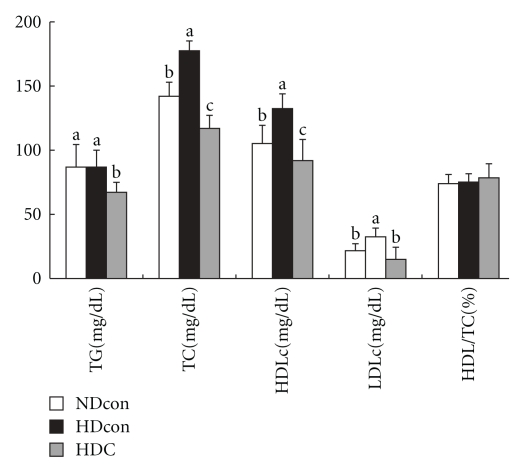
Serum lipid concentrations. Values are means ± SD. Values with different superscript letters (a, b, c) indicate significant differences among groups at *P* < .05 by Duncan's multiple range test.

Dietary and energy intakes and weight changes during the experimental period are shown in Table 3. Dietary intakes were not significantly different among the three groups. Energy intake was significantly higher in HDcon group and HDC group compared with NDcon group but was normalized by Chongkukjang consumption in HDC group (Table 3). Final body weight and epididymal fat accumulation of the HDcon group were significantly higher than in the NDcon group, and the HDC group was significantly lower than the HDcon group. Serum lipid profiles are shown in Figure 1. Serum triglycerides of the HDC group were significantly lower than those of the other two groups. Serum total cholesterol and HDL-cholesterol concentrations were significantly higher in the HDcon group than in the NDcon group, and those of the HDC group were significantly lower than in the NDcon group. LDL-cholesterol concentrations in the HDcon group were significantly higher than in the NDcon group and HDC group.

### 3.2. Chongkukjang Consumption Induced Changes in Gene Expression

**Figure 2:**
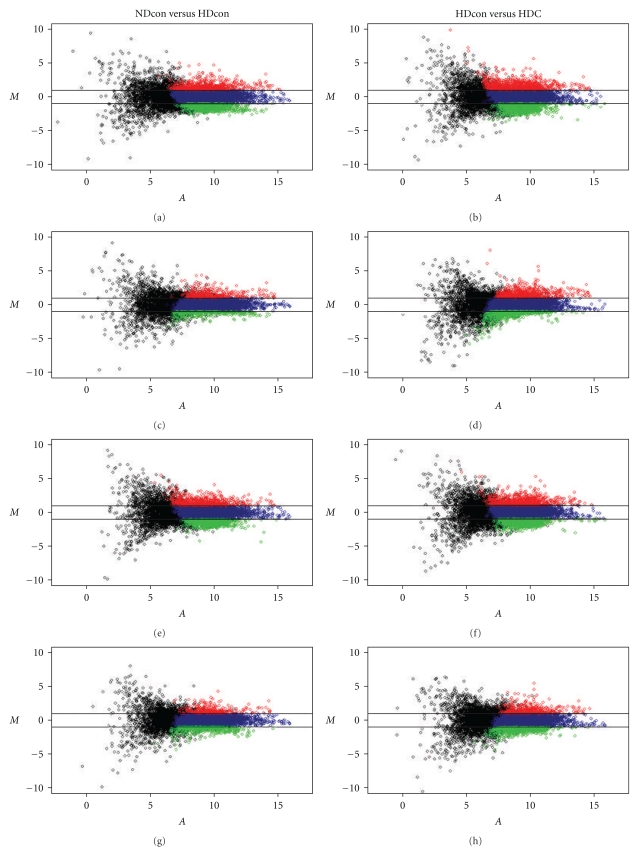
Representative *MA* plots comparing the NDcon versus the HDcon groups and the HDcon versus the HDC groups. *M* represents the log ratio of the average intensities at two fluorescent dyes used to label probes, and *A* represents averaged logarithmic intensity. Broken lines represent a 2-fold change.

The gene expression profile of liver tissues from the NDcon, HDcon, and HDC mice were compared. Only the genes for which changes in mRNA levels were 2.0-fold or more and found to be statistically significant by the SAM method were designated as differentially expressed genes (Figures 1 and 2). By these criteria, 395 genes were found to have significant changes in expression; 220 genes were overexpressed, and 175 genes were underexpressed in liver tissues from HDcon, as compared with the NDcon mice, but were normalized or reversed by Chongkukjang consumption (Table 4). 

The largest number of genes expressed differentially in the HDcon group but normalized by Chongkukjang consumption were those involved in signal transduction, protein synthesis and modification, and metabolism. The genes involved in metabolism, such as carbonic anhydrase 1, squalene epoxidase, lipolysis stimulated lipoprotein receptor, acyl-CoA synthetase long-chain family member 3, 24-dehydrocholesterol reductase, isocitrate dehydrogenase 3 (NAD+), and gamma, were augmented in the HDcon mice, compared to the NDcon mice. These upregulated genes in the HDcon mice were normalized by Chongkukjang consumption (Data not shown). 

The genes involved in protein synthesis and modification such as disintegrin and metallopeptidase domain 10, ubiquitin specific peptidase 25, cathepsin 7, ubiquitin-conjugating enzyme E2N, serine (or cysteine) peptidase inhibitor, clade E, member 2, ring finger protein 103, ubiquitin-conjugating enzyme E2E 3, UBC4/5 homolog (yeast), ubiquitin-conjugating enzyme E2I, and ring finger protein 17 were also increased by at least 2-fold in the HDcon mice and normalized by Chongkukjang consumption. The genes implicated in transcription regulation showed a similar pattern: nuclear factor, erythroid derived 2,-like 1, FBJ osteosarcoma oncogene, c-myc-binding protein, retinoic acid receptor, and gamma. The genes which play roles in signal transduction/apoptosis/cell cycle and transcription regulation also showed similar expression patterns as those described above: Src homology 2 domain-containing transforming protein C1, mitogen-activated protein kinase 13, phospholipase C, delta 1, Src homology 2 domain-containing transforming protein D, cyclin L1, sphingosine kinase 1, mitogen-activated protein kinase 14, cell division cycle 6 homolog (S. cerevisiae), cyclin B1. 

Several genes encoding metabolic enzymes such as acetyl coenzyme A acyltransferase 2 (Acaa2), hydroxysteroid 11-beta dehydrogenase 1, monoglyceride lipase, phytanoyl-CoA hydroxylase, and isocitrate dehydrogenase 3 (NAD^+^) alpha were decreased by at least 2-fold in the HDcon mice and normalized by Chongkukjang consumption. Genes for lipid transporter such as solute carrier family 27 (fatty acid transporter), and member 2 (Slc27a2) and solute carrier family 27 (fatty acid transporter), member 5 (Slc27a5) were also decreased in the HDcon mice and normalized by Chongkukjang consumption. Other genes showing a similar expression pattern included three genes for ATP synthase (ATP synthase, H^+^ transporting mitochondrial F1 complex, beta subunit, ATP synthase, H^+^ transporting, mitochondrial F0 complex, subunit g, ATP synthase, H^+^ transporting, mitochondrial F1 complex, and gamma polypeptide 1), cytochrome c oxidase subunit VIIa polypeptide 2-like and electron transferring flavoprotein, and dehydrogenase.

### 3.3. Confirmation of Differential Expression of Selected Genes by Real-Time RT-PCR Analysis

Changes in gene expression observed by DNA microarray analysis were further confirmed with a small set of known genes by real-time RT-PCR (SYBR Green I), using the same mice liver samples used in the above microarray hybridization. When gene expression profiles obtained by both microarray analysis and RT-PCR were compared, their patterns were very similar with regard to the direction (up- or downregulation) and degree of differences in expression (Table 5). Expression levels of Cpt1, which were not spotted on the microarray or rejected according to microarray technical criteria, were changed by 1.73-fold and 3.41 in the HDcon and HDC mice, respectively, compared with NDcon, when examined using real-time RT-PCR.

## 4. Discussion

To assess the effects of Chongkukjang consumption on high-fat diet-induced obesity in C57BL6J mice, we investigated diet and energy intakes, body fat distribution, and lipid profiles. Body weight was increased by high-fat diet in the HDcon group, but it remained normal in the HDC group. Although diet intake was not significantly different, unsaturated fatty acids (linoleic acid and oleic acid) intake was three times higher in HDC group than HDcon group, and the increased caloric density of the high-fat diet led to significantly higher weight gain compared with the normal diet, which resulted in higher feed efficiency. Thus, mice fed a high-fat diet showed a more rapid growth and greater epididymal fat accumulation than the normal-diet-fed mice. However, the higher weight gain and greater epididymal fat accumulation exhibited by the high-fat diet group were reduced by Chongkukjang consumption. Changes in epididymal fat accumulation reflected body weight changes. These results suggest that Chongkukjang consumption can suppress the increase of weight gain induced by a high-fat diet.

In this study, a high-fat diet significantly increased triglyceride and LDL-cholesterol concentration in serum, which has been shown in other studies [16, 17]. Generally, the LDL-cholesterol level in the blood is considered in determining the malfunction of lipoprotein metabolism, because the LDL-cholesterol level has shown a direct proportional correlation to both coronary heart disease and atherosclerosis [18]. Soybean consists of 40% protein and 20% lipids, with a composition that is nearer to flesh foods and meat, but it also contains high fiber, unsaturated fatty acids (linoleic acid 54% and oleic acid 28%), and no cholesterol [19]. The physiological effects of soybeans may also be partially due to increased excretion of bile acid after ingestion [20], but investigator's opinions on the effects of soy consumption are varied. Chongkukjang was fermented predominantly with *Bacillus *species, as with natto fermentation. Several studies reported that fiber content, vitamin K, and isoflavonoid aglycones % were increased, and bioactive compounds such as polyglutamate were created in Chongkukjang during fermentation [21, 22]. Our results have shown that Chongkukjang supplemented diets improve lipid profiles, reduce epididymal fat deposits, and induce weight loss in high-fat diet-induced obese mice. We conjecture that these results are influenced by complex effects of several physiologically bioactive materials that are created during Chongkukjang fermentation. We expect that Chongkukjang consumption can decrease the occurrence of coronary heart diseases such as arteriosclerosis that are common due to poor dietary habits such as high-fat and high-cholesterol meals.

Because a global analysis of gene expression in response to changes in physiological status appears to be essential for understanding the molecular mechanisms underlying the alterations, we investigated the hepatic transcriptional profiles in a diet-induced obesity mouse model and their alteration by Chongkukjang consumption using a cDNA microarray.

Mammalian tissues have four known types of thiolase which differ in intracellular localization. Cytosolic acetoacetyl-CoA thiolase catalyzes the formation of acetoacetyl-CoA required for cholesterol biosynthesis [23]. Two types of thiolase in the mitochondrial matrix have been identified. One is a mitochondrial acetoacetyl-CoA thiolase, which seems to function in ketone body metabolism [24], and the other is a mitochondrial 3-oxoacyl-CoA thiolase (acetyl-CoA acyltransferase), which catalyzes the last step of the fatty acid *β*-oxidation cycle [23–25]. The Acaa gene encodes an enzyme, acetyl-CoA acyltransferase(3-oxoacyl CoA thiolase), in fatty acid oxidation. Acetyl-CoA acyltransferase generates acyl-CoA and makes fatty acids available to carnitine and Cpt1 to facilitate entry into the fatty acid oxidation pathway.

In a study by Takahashi et al., hepatic Acaa gene expression was upregulated by CLA with a decrease in body weight gain and epididymal white adipose tissue weight [26]. In a study by Doi et al., the lipoprotein lipase (LPL) activator ibrolipim orally administered to rats for 7 days caused a 1.54-fold increase in carnitine palmitoyl transferase II (CptII) mRNA in the carnitine palmitoyl transferase system. Furthermore, ibrolipim caused a 1.47-fold increase in long-chain acyl-CoA dehydrogenase (LCAD) mRNA, a 1.49-fold increase in acetyl-CoA acyltransferase 2 (Acaa2) mRNA, and a 1.24-fold increase in enoyl-CoA hydratase (ECH) mRNA in rats, all of which are liver *β*-oxidation enzymes when compared to the control group [27]. Lipases are serine hydrolases which cleave lipids essential as energy sources (e.g., triacylglycerols) and signaling molecules (2-arachidonoylglycerol) [28, 29]. These enzymes, which play key roles in lipid metabolism and transport, are anchored to the plasma membrane of endothelial cells and are released after intravenous heparin administration—thus the term post heparin plasma lipolytic activity [30]. In this study, we found that Acaa2, Cpt1, and Mgll (monoglyceride lipase) genes were upregulated by Chongkukjang consumption. Cpt1, localized in the outer mitochondrial membrane, catalyzes the formation of long-chain acylcarnitines from their respective CoA esters, committing them to *β*-oxidation in the mitochondrial matrix. In different studies, hepatic Cpt1 expression was increased by exercise or high-fat diet, [31–33], suggesting that control of Cpt1 gene expression is a key feature in the regulation of fatty acid oxidation during exercise [34]. Fat feeding, fasting, induced diabetes, or treatment of rats with peroxisomal/mitochondrial proliferating agents all enhance the capacity for hepatic fatty acid oxidation and increase the mRNA and activity levels of Cpt1 [35, 36]. Under these conditions, the expression of Cpt1 is required to obtain energy from fatty acids, the primary energy substrate. In our precedence study, the level of TG in liver was significantly decreased in Chongkukjang consumption group, and hepatic CPT-I mRNA expression was also 1.9-fold increased in Chongkukjang consumption group compared to nonfermented soybean consumption group (data not shown). These results served to enhance our understanding of the molecular mechanisms underlying regulation of fatty acid metabolism by Chongkukjang consumption.

In this study, Slc27a2 and Slc27a5 genes encoding fatty acid transport proteins (FATPS/solute carrier family 27) were also upregulated by Chongkukjang consumption. Fatty acid transport proteins are integral transmembrane proteins that enhance the uptake of long-chain and very long-chain fatty acids into cells [37, 38]. The FATPs comprise a family of six highly homologous proteins, FATP1-6, which are found in all fatty acid utilizing tissues of the body [39–41]. Therefore, we expect that the fatty acid utilization was increased by Chongkukjang consumption.

Hepatic Phyh gene was downregulated by high-fat diet but was normalized by Chongkukjang consumption. The Phyh gene product, Phytanoyl-CoA hydroxylase (Phyh), catalyzes the conversion of phytanoyl-CoA to 2-hydroxyphytanoyl-CoA, which is the first step in the phytanic acid *α*-oxidation pathway [42]. Fatty acids containing a methyl group at the 3-position cannot be *β*-oxidized directly but first require the oxidative removal of the terminal carboxyl-group in a process called *α*-oxidation. The product of this pathway is a 2-methyl fatty acid which then can undergo *β*-oxidation. Most studies on the mechanism of *α*-oxidation have been performed with phytanic acid (3, 7, 11, and 15-tetramethylhexadecanoic acid) as this fatty acid accumulates in patients suffering from Refsum's disease (RD), a rare inborn error of metabolism. For a long time, it was thought that *α*-oxidation of phytanic acid involved the free fatty acid and not the CoA-ester. Studies by Watkins et al. [43] however, revealed that phytanoyl-CoA is the true substrate for *α*-oxidation. Mihalik et al. [44] showed that the first step in the *α*-oxidation process, the conversion of phytanoyl-CoA into 2-hydroxyphytanoyl-CoA is catalyzed by phytanoyl-CoA hydroxylase. We hypothesize that upregulation of phyh gene by Chongkukjang activates the *α*-oxidation process for phytanic acid oxidation.

Cholesterol accounts for 99% of all sterols in mammals, playing multiple biological roles as a major constituent of membranes, a precursor to numerous signaling molecules, and an inducer of the Hedgehog family of morphogens [45]. However, hypercholesterolemia is a major risk factor for cardiovascular diseases such as atherosclerosis, myocardial infarction, heart attacks, and cerebrovascular diseases [46]. The Dhcr24 gene encodes an enzyme catalyzing the last step of cholesterol biosynthesis, the conversion of desmosterol to cholesterol. In this study, serum total cholesterol concentration was increased by high-fat diet and was normalized in the HDC group by Chongkukjang consumption. We hypothesize that the change in serum total cholesterol concentration was caused by change of hepatic Dhcr24 gene expression.

In summary, we have demonstrated that Chongkukjang consumption has beneficial effects on body weight, epididymal fat accumulation, serum lipid profile and hepatic gene expression in mice fed a high-fat diet. The Chongkukjang consumption decreased weight gain, epididymal fat accumulation, serum triglyceride, total cholesterol, and LDL-cholesterol. In addition, the majority of genes expressed differentially in the high-fat diet group were normalized by Chongkukjang consumption. We propose that Chongkukjang consumption improves serum lipid profiles and body fat accumulation, probably by modulating transcriptional levels of enzymes for utilization of fatty acid.

Therefore, Chongkukjang may prevent obesity induced by long-tem high-fat diet. We expect that our microarray result will provide useful basic data in research that investigates the effect of Chongkukjang consumption on the metabolic disease by diet-induced obesity.

## Figures and Tables

**Figure 3:**
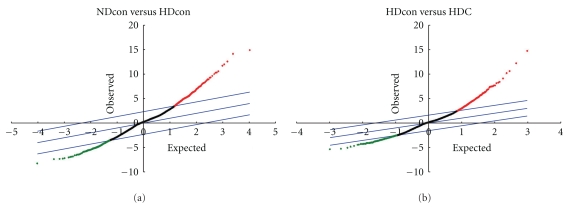
SAM scatter plot of observed relative difference versus the expected relative difference. The genes showing significant difference in expression between the NDcon and the HDcon or the HDcon and the HDC mice were identified. Broken lines represent *δ* = 0.66.

**Table:** (a)

	NDcon	HDcon	High-fat diet	HDC
	*A (10% fat)	*B (45% fat)	*C (60% fat)	C plus CKJ
	1000 g	1000 g	1000 g	600 g
Casein, 80 mesh	189.56	233.06	258.45	155.07
L-Cystine	2.84	3.50	3.88	2.33
Corn starch	298.56	84.83	0.00	0.00
Maltodextrin 10	33.17	116.53	161.53	96.92
Sucrose	331.74	201.36	88.91	53.34
Cellulose, BW200	47.39	58.26	64.61	38.77
Soybena oil	23.70	29.13	32.31	19.38
Lard	18.96	206.84	316.60	189.96
Mineral mix S10026	9.48	11.65	12.92	7.75
DiCalcium phosphate	12.32	15.15	16.80	10.08
Calcium carbonate	5.21	6.41	7.11	4.26
Potassium citrate, 1 H_2_O	15.64	19.23	21.32	12.79
Vitamin mix V10001	9.48	11.65	12.92	7.75
Choline bitartrate	1.90	2.33	2.58	1.55
FD&C yellow dye #5	0.05	—	—	—
FD&C red dye #5	—	0.06	—	—
FD&C blue dye #5	—	—	0.06	0.04
Chongkukjang (CKJ)	0.00	—	—	400

kcal/1000 g	3845.32	4727.61	5242.62	4929.57

*Modified AIN-93 diet (Research Diets, Inc., USA).

**Table:** (b)

	Normal diet	High-fat diet	
	NDcon^(1)^	HDcon^(2)^	HDC^(3)^

Carbohydrates (energy %)	70	35	31
Protein (energy %)	20	20	22
Fat (energy %)	10	45	48
Kcal/g	3.85	4.73	4.93

^(1)^AIN-93 modified diet with 4% fat (10% fat calorie) content. ^(2)^AIN-93 modified diet with 24% fat (45% fat calorie) content. ^(3)^AIN-93 modified high-fat diet (60% fat calorie) plus 40% fermented soybean paste.

**Table 2:** Primers for RT-PCR.

Gene	Sense	Anti-sense
Acaa2	5′-TGTGTCAGAAATGTGCGCTTC-3′	5′-CAAGGCGTATCTGTCACAGTC-3′
Dhcr24	5′-GCACAGGCATCGAGTCATC-3′	5′-GGCACGGCATAGAACAGGTC-3′
Dpagt1	5′-CTCGCTGTTGGGATTCGTG-3′	5′-GCTGAGCTTGTTGAGGTCCTG-3′
Hsd11b1	5′-CTCCAGAAGGTAGTGTCTCGC-3′	5′-CCTTGACAATAAATTGCTCCGCA-3′
Mgll	5′-CGGACTTCCAAGTTTTTGTCAGA-3′	5′-GCAGCCACTAGGATGGAGATG-3′
Phyh	5′-ACTGCCTTCTCCCCGAGATT-3′	5′-CCGGGATGTCTTCTTGCCA-3′
Pon1	5′-TACTGGTGGTAAACCATCCAGA-3′	5′-GCAGCTATATCGTTGATGCTAGG-3′
Prkag1	5′-GAAGCAGTGTTTTGTGGGCAT-3′	5′-ACGTCTCTATCTTGTGCTCCT-3′
Slc27a2	5′-GAGTCGTGGAGGTCTGAAGTC-3′	5′-ACCTTAGGCGATGATGATTGATG-3′
Slc27a5	5′-CTACGCTGGCTGCATATAGATG-3′	5′-CCACAAAGGTCTCTGGAGGAT-3′
Cpt1l	5′-AGAATCTCATTGGCCACCAG-3′	5′-CAGGGTCTCACTCTCCTTGC-3′
*β*-actin	5′-GGGTCAGAAGGACTCCTATG-3′	5′-GATACAATGCCATGTTCAAT-3′

**Table 3:** Body weight and food intake in mice fed experimental diets for 9 weeks.

	Normal diet	High fat diet	
	NDcon	HDcon	HDC
Dietary intake (g/day)	2.40 ± 0.27	2.27 ± 0.30	2.11 ± 0.39
Energy intake (kcal/day)	9.15 ± 1.03^b^	10.69 ± 1.43^a^	10.72 ± 1.79^a^
Initial body weight (g)	22.67 ± 1.14	23.23 ± 1.33	23.99 ± 1.19
Final body weight (g)	27.10 ± 1.44^b^	30.02 ± 2.47^a^	27.96 ± 1.73^b^
Epididymal fat (g/b · w (%))	2.21 ± 0.58^b^	3.54 ± 1.32^a^	1.84 ± 0.18^c^

Values are means ± SD. Values with different superscript letters (a, b, c) indicate significant differences among groups at *P* < .05 by Duncan's multiple range test.

**Table 4:** The numbers of genes differentially expressed in HDcon Mice but normalized or reversed by Chongkukjang consumption (HDC)^(1)^.

Molecular function	Increase	Decrease	Total
Metabolism	19	34	53
Defense/stress/inflammation responses	0	13	13
Signal transduction/apoptosis/cell cycle	66	31	97
Transcription regulation	28	22	50
Protein synthesis and modification	24	19	43
Transport	23	20	43
Cellular adhesion/cytoskeleton/trafficking	11	5	16
Chromosome remodeling	14	6	20
DNA replication	5	4	9
RNA processing	10	2	12
Microtubule-based movement	7	4	11
Unclassified	13	15	28

Total	220	175	395

^(1)^Data represent a summary of results of six independent hybridizations. In three of six replicated experiments, the labeling of HDcon and NDcon samples was reversed to compensate for any nonlinearity in the emission signal intensity response curve for each fluorophore. Genes designated as differentially expressed in the HDcon mice were those for which the normalized signal increased or decreased at least 2.0-fold and detected as significant change by SAM method.

**Table 5:** Comparison between cDNA microarray analysis and real-time RT-PCR.

Genbank No.	Gene	cDNA microarray		Real time RT-PCR	
		HDcon	HDC	HDcon	HDC
BG085346	Acetyl-Coenzyme A acyltransferase 2 (Acaa2)	−4.18	*↔* ^(1)^	0.28^(2)^	0.94
AA277495	Carnitine palmitoyltransferase 1a, liver (Cpt1a)	ND^(3)^	ND	1.73	3.41
BF457090	24-dehydrocholesterol reductase (Dhcr24)	2.1	*↔*	1.45	0.78
BG063933	Dolichyl-phosphate (UDP-N-acetylglucosamine) acetylglucosaminephosphotransferase 1 (Dpagt1)	2.3	*↔*	3.02	1.91
AA023077	Hydroxysteroid 11-beta dehydrogenase 1 (Hsd11b1)	−8.95	*↔*	0.23	1.14
AI835105	Monoglyceride lipase (Mgll)	−8.99	*↔*	0.29	0.62
W82212	Phytanoyl-CoA hydroxylase (Phyh)	−5.78	*↔*	0.37	1.21
AI893897	Paraoxonase 1 (Pon1)	2.0	*↔*	1.68	0.69
AA259329	Protein kinase, AMP-activated, gamma 1 noncatalytic subunit (Prkag1)	2.2	*↔*	2.20	1.41
AA108401	Solute carrier family 27 (fatty acid transporter), member 2 (Slc27a2)	−4.06	*↔*	0.25	0.86
AA254935	Solute carrier family 27 (fatty acid transporter), member 5 (Slc27a5)	−11.73	*↔*	0.25	0.83

^(1)^The symbol double-headed arrow (*↔*) denotes that there is no significant difference when gene expression in HDcon or HDC groups was compared with the NDcon group. ^(2)^Data are expressed as fold changes, normalized to *β*-actin mRNA expression, where the values for the NDcon mice were set at 1.00. The analyses were performed in duplicate. ^(3)^ND, no detected.
